# Computational Prediction of Chiral Iron Complexes for Asymmetric Transfer Hydrogenation of Pyruvic Acid to Lactic Acid

**DOI:** 10.3390/molecules25081892

**Published:** 2020-04-20

**Authors:** Wan Wang, Xinzheng Yang

**Affiliations:** 1 State Key Laboratory for Structural Chemistry of Unstable and Stable Species, Institute of Chemistry Chinese Academy of Sciences, Beijing 100190, China; ww2016@iccas.ac.cn; 2 University of Chinese Academy of Sciences, Beijing 100049, China; 3 Beijing National Laboratory for Molecular Sciences, CAS Research/Education Center for Excellence in Molecular Sciences, Beijing 100190, China

**Keywords:** asymmetric transfer hydrogenation, density functional theory, bifunctional catalyst

## Abstract

Density functional theory calculations reveal a formic acid-assisted proton transfer mechanism for asymmetric transfer hydrogenation of pyruvic acid catalyzed by a chiral Fe complex, FeH[(*R*,*R*)-BESNCH(Ph)CH(Ph)NH_2_](*η*^6^-*p*-cymene), with formic acid as the hydrogen provider. The rate-determining step is the hydride transfer from formate anion to Fe for the formation and dissociation of CO_2_ with a total free energy barrier of 28.0 kcal mol^−1^. A series of new bifunctional iron complexes with *η*^6^-*p*-cymene replaced by different arene and sulfonyl groups were built and computationally screened as potential catalysts. Among the proposed complexes, we found **1_g_** with *η*^6^-*p*-cymene replaced by 4-isopropyl biphenyl had the lowest free energy barrier of 26.2 kcal mol^−1^ and excellent chiral selectivity of 98.5% *ee*.

## 1. Introduction

**Scheme 1:**
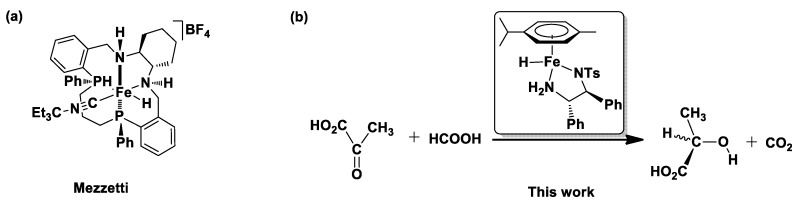
Mezzetti’s N_2_P_2_ macrocyclic iron complex (**a**) and the asymmetric transfer hydrogenation (ATH) of pyruvic acid to lactic acid with HCOOH as hydrogen donor catalyzed by iron hydride complex in this work (**b**).

The asymmetric hydrogenation of prochiral unsaturated ketones is an important process for the synthesis of enantiopure alcohols for use in the pharmaceutical, agrochemical, fragrance, and flavor industries [1,2,3]. As a promising alternative to reduce the use of gaseous hydrogen for the production of enantiopure alcohols, asymmetric transfer hydrogenation (ATH) from low-cost and safe hydrogen donors, usually NH moieties, to ketones catalyzed by organometallic complexes has attracted increasing attention in the past two decades [4,5,6,7,8]. A series of particularly efficient catalytic systems with high reactivity and enantioselectivity for ATH are Noyori and co-workers’ chiral bifunctional Ru and Ir complexes, which are stabilized by soft ligands (*η*^6^-arene [9,10] or phosphine [11,12]) and contain at least one hard NH electron donor that activates or directs the ketone toward hydride attack. However, most of the reported efficient catalysts for this reaction were based on noble metals such as Ru [13,14], Os [2,15], Rh [16,17], or Ir [6,18]. Base-metal catalysts, especially the cheaper and less-toxic iron catalyst with comparable activity, would be desirable in asymmetric catalysis. In 2008, Morris’ group pioneered the field of Fe(II) catalysis with tetradentate PNNP ligands for the asymmetric hydrogenation of polar bonds and attained an excellent turnover frequency (TOF) of 907 h^−1^ but less activity on the enantioselectivity (29% *ee*) [19]. In 2015, Gao and co-workers [20] reviewed a series of iron, cobalt, and nickel catalysts containing novel chiral aminophosphine ligands for ATH or AH of ketones. Among those base metal catalysts, the P_2_N_4_ macrocycles in connection with Fe(0) precursors exhibited high yield (up to 96%) with extraordinary enantioselectivities (up to 99% *ee*) at 65 °C [20]. Mezzetti’s group showed that stable, diamagnetic Fe(II) complexes bearing (NH)_2_P_2_ macrocyclic ligands catalyzed the ATH of a broad scope of ketones with up to 99.5% yield and excellent enantioselectivity (up to 98% *ee*) at 75 °C [21,22]. The molecules containing base-labile stereocenters would easily racemize under basic conditions [23]. They furthermore exploited the dicationic (NH)_2_P_2_ macrocyclic ligands to develop the corresponding iron hydride complex, which possessed the active H-Fe-N-H motif for the base-free ATH of ketones in a yield up to 99% with an enantioselectivity up to 99% at 50 °C (Scheme 1a) [24].

Although promising progress in the iron-catalyzed enantioselective reduction of ketones has been achieved, most of the reported catalysts contained air and moisture-sensitive phosphine ligands, which require rather rigid conditions in the synthesis of catalyst. The development of highly active phosphine-free iron catalyst is therefore highly desirable. Herein, we proposed a series of chiral Fe complexes based on previously reported Ru and Os complexes with similar arene and Tsdiamine ligands [2,15,25] and computationally evaluated their catalytic activities and enantioselectivities for ATH of pyruvic acid (PA) to lactic acid using the density functional theory (DFT). As shown in Scheme 1b, the ATH reaction catalyzed by FeH[(*R*,*R*)-BESNCH(Ph)CH(Ph)NH_2_](*η*^6^-*p*-cymene)(BES, benzenesulfonyl) [26] had formic acid (FA) as the hydrogen donor.

## 2. Results and Discussion

**Figure 1:**
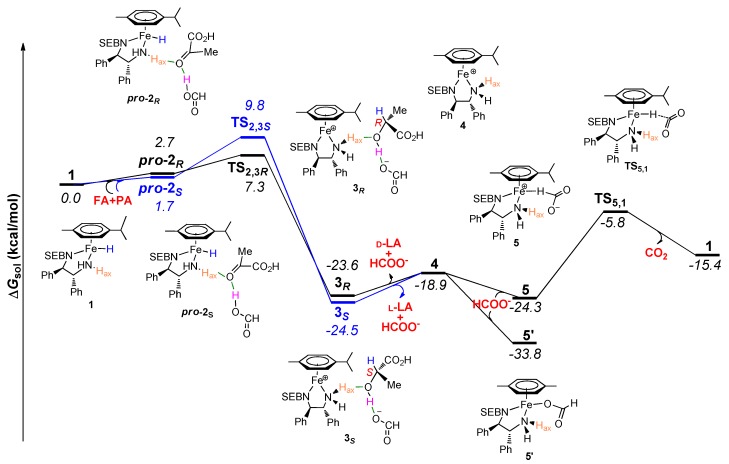
Free energy profile for the ATH of pyruvic acid (PA) catalyzed by **1** with the participation of one formic acid (FA) molecule. The spin-states of all intermediates and transition states are triplet.

**Figure 2:**
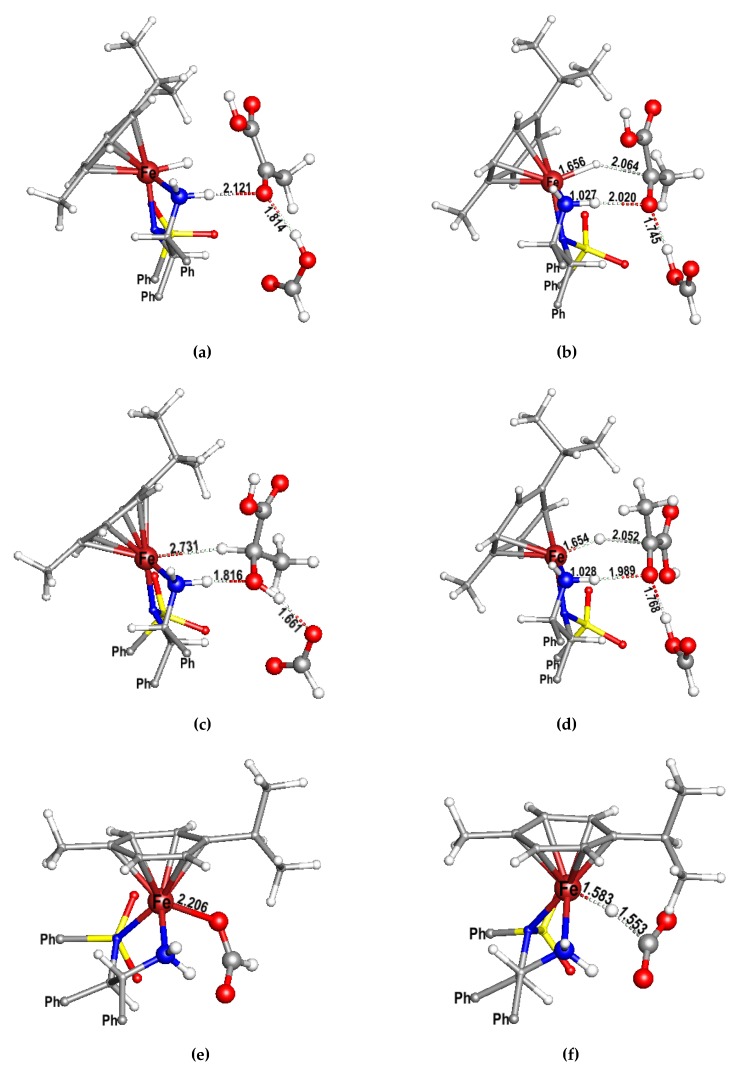
Optimized structures of (**a**) ***pro*-2*_R_***, (**b**) **TS_2,3-*R*_** (227*i* cm^−1^), (**c**) **3*_R_***, (**d**) **TS_2,3-*S*_** (140*i* cm^−1^), (**e**) **5′**, and (**f**) **TS_5,1_** (520*i* cm^−1^). Bond lengths are in Å. Phenyl groups are omitted for clarity.

As illustrated in Figure 1, our DFT calculations revealed a proton-coupled hydride transfer mechanism for the ATH of PA catalyzed by FeH[(*R*,*R*)-BESNCH(Ph)CH(Ph)NH_2_]-(*η*^6^-*p*-cymene) (**1**) with formic acid as the hydrogen source. The optimized structures of key intermediates and transition states are displayed in Figure 2.

The catalytic cycle began with the approach of a PA molecule to **1** from its *Re*-face for the formation of a 2.7 kcal mol^−1^ less-stable prochiral intermediate ***pro*-2*_R_***. The iron hydride and FA proton in ***pro*-2*_R_*** simultaneously transferred to the carbonyl group in PA via transition state **TS_2,3-*R*_** to form a d-lactic acid (d-LA) molecule in **3*_R_***, which is a stable solvent-shared ion pair [27] between the cationic Fe complex and the formate anion mediated by d-LA. The C‒H∙∙∙Fe distance of 2.73 Å in **3*_R_*** suggested a very weak interaction between Fe and the transferred hydride. This process was 26.3 kcal mol^−1^ exothermic and accompanied by the moving of the Fe‒N‒N plane to a position perpendicular to the arene ring on top of Fe. The dissociation of d-LA and formate anion from **3*_R_*** for the formation of cationic Fe complex **4** was a 4.7 kcal mol^−1^ uphill step. The formation of l-lactic acid (l-LA) went through a similar process but had slightly different relative energies. The enantio-determining step (EDS) for the formation of l-LA (**TS_2,3-*S*_**) was 3.2 kcal mol^−1^ higher than that of **TS_2,3-*R*_**, which suggested a chiral selectivity of 99.1% *ee*. The dissociated formate then came back to **4** and formed a slightly more stable intermediate **5** with a hydride coordinated to the Fe center. The transition state for hydride transfer from formate to Fe (**TS_5,1_**) for the formation and release of CO_2_ was 18.5 kcal mol^−1^ higher than that of **5**. Alternatively, the dissociated formate anion could have also coordinated to the Fe center with one of its oxygen atoms and formed a much more stable complex **5′** with a strong Fe‒O bond (2.21 Å). According to the energy span model [28], **5′** and the **TS_5,1_** were the rate-determining states (RDS) with a free energy barrier of 28.0 kcal mol^−1^.

**Scheme 2:**
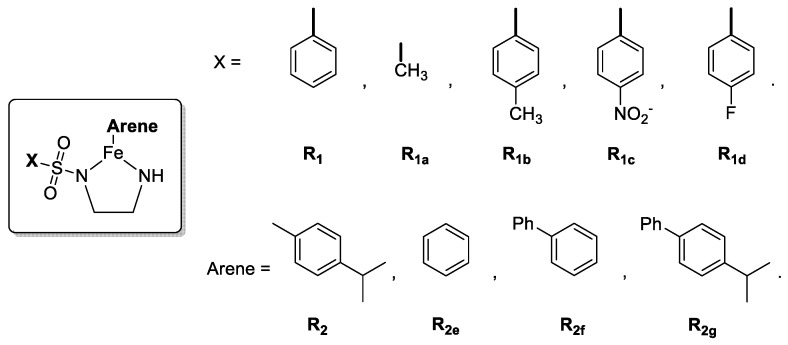
New Fe complexes with methyl or various arene ligands.

Noyori’s work further shows that the application of ligand ArSO_2_ group [29] and the electron donor alkyl group (CH_3_, *i*-C_3_H_7_) on the *η*^6^-arene [30] are important for maintaining catalytic activity and enantioselectivity. In the hope of discovering more active and enantioselective iron catalysts, we investigated the influence of substituents on the sulfonyl and arene groups for catalytic ability and enantioselectivity. As shown in Scheme 2, we built a series of asymmetric iron complexes **1_a_**-**1_g_** (Table 1) by replacing the phenyl ligand in the sulfonyl group with methyl (**R_1a_**), 4-methylphenyl (**R_1b_**), 4-nitrophenyl (**R_1c_**), and 4-fluorophenyl (**R_1d_**) and replacing the *η*^6^-*p*-cymene ligand with phenyl (**R_2e_**), biphenyl (**R_2f_**), and 4-isopropyl biphenyl (**R_2g_**). Then, we computationally examined the catalytic activities and enantioselectivities of complexes **1_a_**-**1_g_** for ATH of PA by comparing the relative free energies of their EDSs (**TS_2,3-*R*_**→**TS_2,3-*S*_**) and RDSs (**5′**→**TS_5,1_**).

As is shown in Table 1, the rate-determining barriers of newly proposed Fe complexes **1_a_**, **1_b_**, **1_d_**, **1_e_**, and **1_g_** were slightly lower than that of **1**. The lowest barrier belonged to **1_g_** at 26.2 kcal mol^−1^ while maintaining an excellent enantioselectivity of 2.9 kcal mol^−1^. Complexes **1_b_**, **1_d_,** and **1_e_,** with higher enantioselectivities of 3.8, 4.1, and 4.9 kcal mol^−1^ respectively, also had lower RDS barriers than those of **1**. The above results suggest that the substituents on both the sulfonyl group and the arene group had significant impact on the catalytic activities and enantioselectivities of proposed iron complexes for asymmetric hydrogenation of PA.

**Figure 3:**
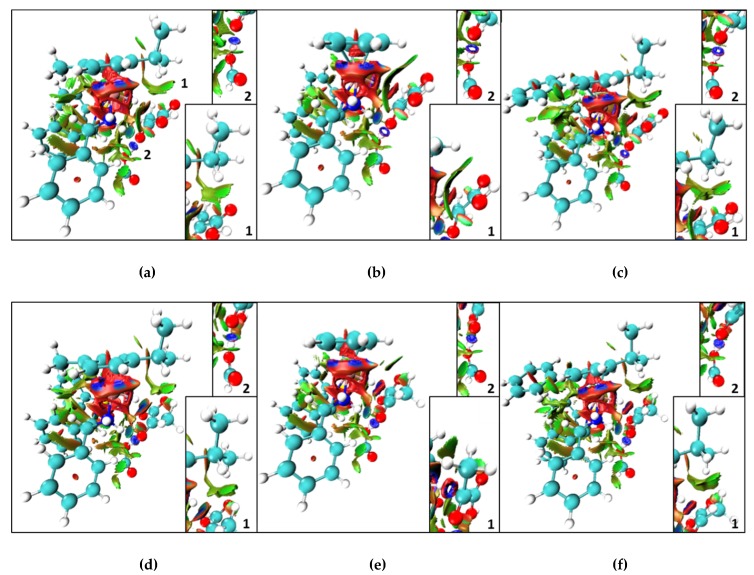
NCI plots for transition states (**a**) **TS_2,3-*R*_**, (**b**) **TS_2,3e-*R*_**, (**c**) **TS_2,3g-*R*_**, (**d**) **TS_2,3-*S*_**, (**e**) **TS_2,3e-*S*_**, and (**f**) **TS_2,3g-*S*_**. The important stabilizing interactions are marked with numbers and highlighted in the box with a zoomed-in view. Color code: red, repulsive; green, weak attractive; blue, attractive.

The enantio-determining transfers of the hydride on iron and the proton on FA to the carbonyl group of PA proceeded through an outer-sphere mechanism. In order to understand the weak interaction between unsaturated compound, catalyst, and solvent molecules, we performed a noncovalent interaction (NCI) analysis [31] for transition states **TS_2,3-*R*_** and **TS_2,3-*S*_**. As shown in Figure 3, we found a stronger attraction between C-H protons of the *η*^6^-*p*-cymene group and the carboxyl group of PA in **TS_2,3-*R*_**, which is highlighted in position 1 with a zoomed-in view and suggests a stronger C-H∙∙∙π hydrogen bond. In addition, strong hydrogen bonds between the oxygen of carbonyl group in PA and the hydroxyl hydrogen in FA at **TS_2,3-*R*_** and **TS_2,3-*S*_** are highlighted as insets labeled 2 with a zoomed-in view. We also compared the NCI plots of the transition states of the proposed complexes with better selectivity or activity, including **TS_2,3e-*R*_**, **TS_2,3e-*S*_**, **TS_2,3g-*R*_**, and **TS_2,3g-*S*_**. As a significant stabilizing factor, the C-H∙∙∙π attraction in **TS_2,3e-*R*_** was much stronger than that in **TS_2,3e-*S*_** and thus led to better selectivity.

## 3. Computational Methods

All DFT calculations were performed using the Gaussian 09 suite of ab initio programs [32] for a hybrid meta-GGA level M06 functional [33] in conjugation with all-electron 6-31G(d) basis set [34] for H, C, N, O, S, and F atoms. Stuttgart relativistic effective core potential basis sets were used for the Fe (ECP10MDF) atom [35]. All structures were fully optimized in formic acid (*ε* = 51.1) using the integral equation formalism polarizable continuum solvation model (IEFPCM) [36] with the SMD radii [37] for solvent effect corrections. An ultrafine grid (99,590) was used for numerical integrations. The ground states of intermediates were confirmed as triplets through comparison with their low-spin analogs. Thermal corrections were calculated within the harmonic potential approximation on optimized structures under 298.15 K and 1 atm pressure. Unless otherwise noted, the relative energies reported in the text are Gibbs free energies with solvent effect corrections. The optimized structures were confirmed to have no imaginary vibrational mode for all equilibrium structures and only one imaginary vibrational mode for each transition state. Atomic coordinates of all optimized structures (XYZ) and Evaluation of basis sets can be found in Supplementary Materials. Transition states were further characterized by intrinsic reaction coordinate (IRC) calculations to affirm that the correct stationary points were connected. The 3D molecular structures were drawn using the JIMP2 molecular visualizing and manipulating program [38]. NCI analysis was performed using the Multiwfn program [39] for electronic structure analysis and the Virtual Molecular Dynamics (VMD) program [40] for visualization. Computationally enantiomeric ratio was obtained based on Equation (1) [41]:

(1) $$E=\frac{e^{-\frac{\Delta \Delta G}{RT}}-1}{e^{-\frac{\Delta \Delta G}{RT}}+1}\times 100\%$$

## 4. Evaluation of Ground State Spin Multiplicity

In order to find out correct spin multiplicities of the ground states of the Fe complexes in the catalytic reaction, we optimized the structures of all intermediates with singlet and triplet spins using the methods described above. As shown in Table 2, all singlet states were significantly more stable than the corresponding triplet states. Therefore, we believe the ATH of PA reaction catalyzed by **1** went through a high-spin pathway.

## 5. Conclusions

In summary, our DFT study of the asymmetric transfer hydrogenation of pyruvic acid to lactic acid enantiomers catalyzed by bifunctional Fe complex revealed a formic acid-assisted proton-coupled hydride transfer mechanism. The formation of d-lactic acid was 2.5 kcal mol^−1^ more favorable than the formation of l-lactic acid with complex **1** as the catalyst. The rate-determining step in the reaction catalyst by **1** was the regeneration of the catalyst with the formation and release of CO_2_ with a total free energy barrier of 28.0 kcal mol^−1^. We also built and computationally examined a series of chiral Fe complexes as potential catalysts for ATH of PA and found that Fe complexes with substituents 4-methylphenyl (**1_b_**) or 4-fluorophenylphenyl (**1_d_**) on the sulfonyl group, as well as with substituents 4-isopropyl biphenyl (**1_e_**) instead of *η*^6^-*p*-cymene, had potentially better performance than **1** in both catalytic activity and chiral selectivity. Among the proposed iron complexes, **1_g_** had the lowest free energy barrier of 26.2 kcal mol^−1^ and computationally chiral selectivity of 98.5% *ee*. Our findings not only provide promising iron complexes for transfer hydrogenation of pyruvic acid, they also pave the way for developing efficient asymmetric catalysts for ATH reactions, as the participation of solvent molecules might be critical for low-barrier proton and hydride transfers.

## Figures and Tables

**Table 1:** Relative free energies between EDSs to lactic acid enantiomers (**TS_2,3-*R*_** and **TS_2,3-*S*_**) and the rate-determining states (RDSs) (**5′** and **TS_5,1_**) in catalytic cycles.

Complexes	X	Arene	TS_2,3-*R*_→TS_2,3-*S*_	5′→TS_5,1_
			Δ*G* (kcal mol^−1^)	
**1**	**R_1_**	**R_2_**	3.2	28.0
**1_a_**	**R_1a_**	**R_2_**	0.6	27.5
**1_b_**	**R_1b_**	**R_2_**	3.8	27.9
**1_c_**	**R_1c_**	**R_2_**	3.0	28.8
**1_d_**	**R_1d_**	**R_2_**	4.1	27.5
**1_e_**	**R_1_**	**R_2e_**	4.9	27.8
**1_f_**	**R_1_**	**R_2f_**	3.7	28.5
**1_g_**	**R_1_**	**R_2g_**	2.9	26.2

**Table 2:** Absolute and relative free energies of singlet and triplet states of the intermediates in the ATH of pyruvic acid catalyzed by **1**.

Complexes	Singlet (a.u.)	Triplet (a.u.)	Δ*G* (kcal mol^−1^)
**1**	−1944.102697	−1944.118152	−9.7
**2_R_**	−2475.926296	−2475.940345	−8.8
**2_S_**	−2475.924832	−2475.941943	−10.7
**3_R_**	−2475.943887	−2475.982223	−24.1
**3_S_**	−2475.946197	−2475.983624	−23.5
**4**	−1943.384036	−1943.421081	−23.2
**5**	−2132.591160	−2132.625404	−21.5
**5′**	−2132.620516	−2132.640419	−12.5

## Supplementary Materials

The following are available online. Atomic coordinates of all optimized structures (XYZ) and Evaluation of basis sets.
