# Supplementary material for: Computational Prediction of Chiral Iron Complexes for Asymmetric Transfer Hydrogenation of Pyruvic Acid to Lactic Acid
Source: Molecules. 2020 Apr 20;25(8):1892. doi: 10.3390/molecules25081892 (PMC7221593; doi:10.3390/molecules25081892)
Supplement: Supplementary file 1 [file molecules-25-01892-s001.zip › molecules-746663.R1_SI.pdf]

Supplementary Information

Computational prediction of chiral iron complexes for asymmetric transfer hydrogenation of pyruvic acid to lactic acid

Wan Wang [1,2,3] and Xinzheng Yang[1,2,3,*]

[1] State Key Laboratory for Structural Chemistry of Unstable and Stable Species, Institute of Chemistry Chinese Academy of Sciences, Beijing 100190, China
[2] University of Chinese Academy of Sciences, Beijing 100049, China
[3] Beijing National Laboratory for Molecular Sciences, CAS Research/Education Center for Excellence in Molecular Sciences, Beijing 100190, China
* Correspondence: xyang@iccas.ac.cn

1. Evaluation of basis set

In order to evaluate the influential of the basis set superposition errors (BSSE) on enantio-determining step (EDS) of our iron catalytic system, we calculated singlet point energies of the enantio-determining transition states **TS**$_{2,3\text{-}\boldsymbol{R}}$ and **TS**$_{2,3\text{-}\boldsymbol{S}}$ with extended basis sets (def2-TZVP for main group and def2-QZVP for Fe)[1-3]. The absolute free energies are obtained by adding the free energy corrections calculated by using small basis sets (6-31G(d)/ECP10MDF) to the corresponding electronic energies calculated by using extended basis sets (def2-TZVP/def2-QZVP):

$$G_{extended} = E_{extended} + G_{correction}(small\ basis\ set)$$

As shown in Table S1, the free energy difference between **TS**$_{2,3\text{-}\boldsymbol{R}}$ and **TS**$_{2,3\text{-}\boldsymbol{S}}$ with extended basis set is 3.3 kcal mol^{-1}, which is only 0.1 kcal mol^{-1} larger than the relative free energy with small basis set. Such results indicate that the BSSE of 6-31G(d)/ECP10MDF basis sets is negligible for this iron based catalytic system.

Table S1. Absolute and relative energies of **TS**$_{2,3\text{-}\boldsymbol{R}}$ and **TS**$_{2,3\text{-}\boldsymbol{S}}$ with different basis set.

Basis set	**TS**$_{2,3\text{-}\boldsymbol{R}}$ (a.u.)	**TS**$_{2,3\text{-}\boldsymbol{S}}$ (a.u.)	ΔG (EDS) (kcal mol^{-1})
6-31G(d)/ECP10MDF	–2475.934071	–2475.929023	3.2
def2-TZVP/def2-QZVP	–3616.541443	–3616.536225	3.3

Reference:

1. Pritchard, B. P.; Altarawy, D.; Didier, B.; Gibson, T. D.; Windus, T. L., New basis set exchange: An open, up-to-date resource for the molecular sciences community. *J. Chem. Inf. Model.*, **2019,** 59, 4814-4820.
2. Schuchardt, K. L.; Didier, B. T.; Elsethagen, T.; Sun, L.; Gurumoorthi, V.; Chase, J.; Li, J.; Windus, T. L., Basis set exchange: A community database for computational sciences. *J. Chem. Inf. Model.*, **2007,** 47, 1045-1052.
3. Feller, D., The role of databases in support of computational chemistry calculations. *J. Comput. Chem.,* **1996,** 17, 1571-1586.
